# Regulation of Hypoxic–Adenosinergic Signaling by Estrogen: Implications for Microvascular Injury

**DOI:** 10.3390/ph16030422

**Published:** 2023-03-10

**Authors:** Jessica Cassavaugh, Nada Qureshi, Eva Csizmadia, Maria Serena Longhi, Robina Matyal, Simon C. Robson

**Affiliations:** Department of Anesthesia, Critical Care and Pain Medicine, Beth Israel Deaconess Medical Center, Boston, MA 02215, USA

**Keywords:** purinergic signaling, estrogen, adenosine, microvascular, females, hypoxia

## Abstract

Loss of estrogen, as occurs with normal aging, leads to increased inflammation, pathologic angiogenesis, impaired mitochondrial function, and microvascular disease. While the influence of estrogens on purinergic pathways is largely unknown, extracellular adenosine, generated at high levels by CD39 and CD73, is known to be anti-inflammatory in the vasculature. To further define the cellular mechanisms necessary for vascular protection, we investigated how estrogen modulates hypoxic–adenosinergic vascular signaling responses and angiogenesis. Expression of estrogen receptors, purinergic mediators inclusive of adenosine, adenosine deaminase (ADA), and ATP were measured in human endothelial cells. Standard tube formation and wound healing assays were performed to assess angiogenesis in vitro. The impacts on purinergic responses in vivo were modeled using cardiac tissue from ovariectomized mice. CD39 and estrogen receptor alpha (ERα) levels were markedly increased in presence of estradiol (E2). Suppression of ERα resulted in decreased CD39 expression. Expression of ENT1 was decreased in an ER-dependent manner. Extracellular ATP and ADA activity levels decreased following E2 exposure while levels of adenosine increased. Phosphorylation of ERK1/2 increased following E2 treatment and was attenuated by blocking adenosine receptor (AR) and ER activity. Estradiol boosted angiogenesis, while inhibition of estrogen decreased tube formation in vitro. Expression of CD39 and phospho-ERK1/2 decreased in cardiac tissues from ovariectomized mice, whereas ENT1 expression increased with expected decreases in blood adenosine levels. Estradiol-induced upregulation of CD39 substantially increases adenosine availability, while augmenting vascular protective signaling responses. Control of CD39 by ERα follows on transcriptional regulation. These data suggest novel therapeutic avenues to explore in the amelioration of post-menopausal cardiovascular disease, by modulation of adenosinergic mechanisms.

## 1. Introduction

Microvascular or small vessel disease impacts small arterioles and capillary beds, whose primary purpose is the regulation of blood flow to match oxygen requirements and metabolic demands. Microvascular injury contributes to many common pathologies including heart failure, pulmonary hypertension, diabetes mellitus, and cerebrovascular diseases such as stroke [1,2]. The coronary microvasculature is particularly important for maintaining adequate oxygen extraction and therefore myocardial oxygen supply, while also removing metabolic waste. Injury to this vascular bed, as can occur concurrently with atherosclerosis, metabolic syndrome, and other macro-vasculature conditions, results in vascular remodeling, autonomic dysregulation, and endothelial dysfunction [2,3].

Pre-menopausal women are relatively protected from the development of microvascular diseases, likely due to the beneficial effects of estrogens on the endothelium [4]. However, post menopause, women are at a significantly increased risk for developing heart disease, specifically heart failure with preserved ejection fraction (HFpEF), a condition strongly associated with microvascular dysfunction [5,6,7,8,9]. The effects of estrogen on the cardiovascular system are multifaceted ranging from regulation of vascular tone, mitochondrial function, and angiogenesis to inhibition of fibrosis and pathologic remodeling. The estrogen receptor (ER) is a member of the steroid/nuclear receptor family that is encoded by two genes, alpha and beta. The canonical pathway is well described; following ligand binding, ER translocates to the nucleus where it functions either as a transcription factor binding directly to target genes or indirectly through binding to and stabilizing of a second transcription factor [10]. Non-nuclear ER signaling is less well characterized but is thought to occur at the cell membrane [11]. Both mechanisms are involved in reducing ROS, increasing NO, and promoting an overall anti-inflammatory phenotype. 

Extracellular nucleotides facilitate inflammation in the vasculature through interactions with ATP receptors [12]. Purinergic signaling is integral in the regulation of inflammation through hydrolysis of pro-inflammatory ATP to anti-inflammatory adenosine. Several key regulatory proteins are involved including CD39, CD73, adenosine receptors (ADORA), and equilibrative nucleoside transporters (ENT), which function as adenosine transport channels on the cell membrane [13,14]. Hydrolysis of ATP is a two-step process beginning with hydrolysis of ATP/ADP to AMP via the cell membrane ectonucleotidase, CD39. AMP is next hydrolyzed to adenosine by a second membrane bound ecto-5′-nucleotidase, CD73 [15]. Extracellular adenosine interacts with ADORA and initiates various downstream effects or is transported directly intracellular via ENT. Production of adenosine is protective to the microvasculature through the promotion of wound healing and angiogenesis along with the inhibition of platelet aggregation and neutrophil-mediated endothelial injury [13]. 

Previous work has demonstrated Erα-mediated impacts on CD39 expression in immune cells. In an autoimmune hepatitis model, ERα was shown to bind to the CD39 promoter of T cells [16]. However, regulation of the purinergic pathway by estrogen in the vasculature and with exposure to hypoxia remains widely uncharacterized. 

The aim of this study was to therefore investigate the links between hypoxia, purinergic signaling, and estrogen as these relate specifically to the vascular endothelium and the heart. Herein, we report on the estrogen-mediated upregulation of CD39 in hypoxia resulting in positive enhancement of adenosinergic signaling and angiogenesis. This novel regulation of CD39 appears protective for cardiovascular and, more expressly, microvascular function and health. 

## 2. Results

### 2.1. Hypoxia Regulates Estrogen Receptor Alpha Expression

**Figure 1:**
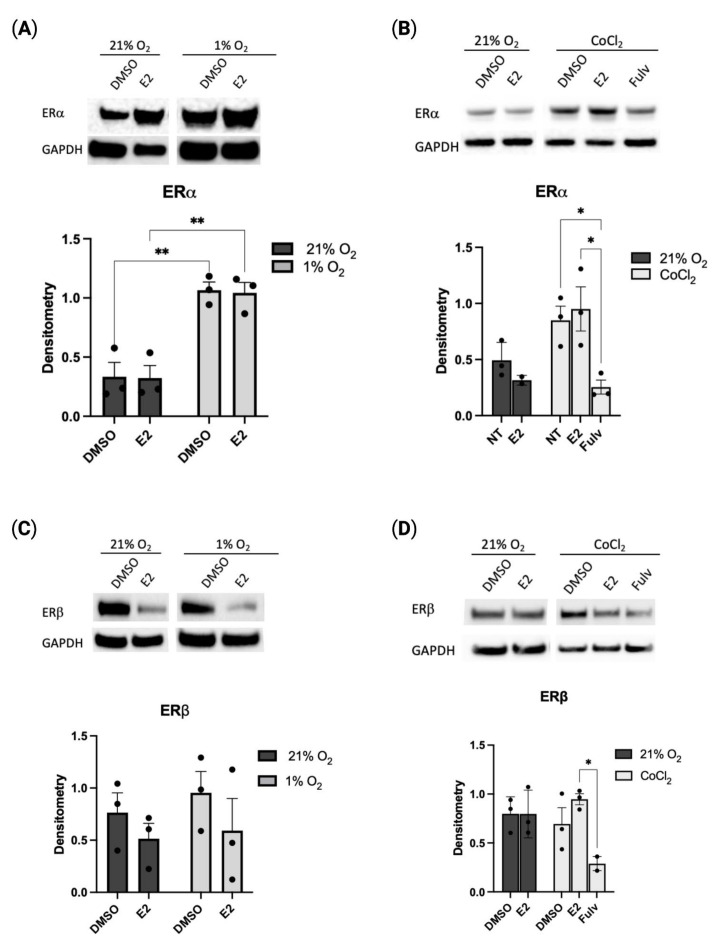
Hypoxia increases expression of ERα in HUVECs. ERα (**A**) or ERβ (**C**) protein expression following 21% oxygen or 1% oxygen in presence of estradiol (E2 1 µM). (**B**,**D**). Expression of ERα (**B**) or ERβ (**D**) following 21% oxygen or Cobalt Chloride (CoCl_2_ 1 µM) treatment in presence of estradiol (E2 1 µM) or fulvestrant (Fulv, 1 µM) for 24 h. Protein expression quantified with densitometry. Data represent the mean ± SEM from three independent experiments (black circles). * *p* < 0.05, ** *p* < 0.01 using one-way ANOVA with post hoc Tukey’s test.

Prior studies have shown regulation of estrogen receptor beta (ERβ) by hypoxia inducible factor 1 alpha (HIF-1α), however, less is known about the regulation of estrogen receptor alpha (ERα) in hypoxia [17]. Following exposure to either 1% oxygen or cobalt chloride, a hypoxic mimetic, the expression of ERα in HUVECs was found to be significantly elevated when compared to normoxic controls. The increase in ERα expression was seen regardless of estradiol (E2) exposure (Figure 1A,B). No changes were observed in ERβ expression in hypoxia (Figure 1C,D). Treatment with the estrogen receptor inhibitor, fulvestrant, prevented any increase in ER expression (Figure 1B,D). HIF-1α expression was found to be elevated in hypoxia following exposure to E2 (Figure S1 (Supplementary Materials)). These findings suggest ERα, not ERβ, is regulated in hypoxic endothelial cells in an estradiol-independent manner.

### 2.2. Estradiol Positively Regulates CD39 Levels in Hypoxia

**Figure 2:**
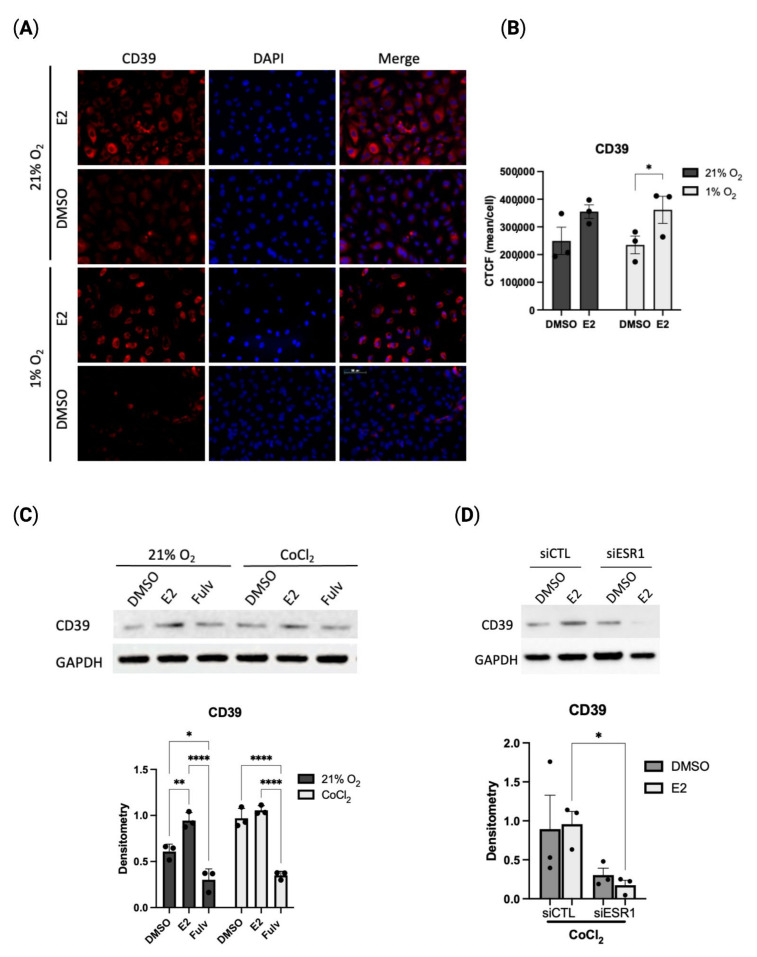
Estradiol regulates CD39 expression in hypoxic HUVECs. (**A**) Representative immunofluorescent images (CD39 red. DAPI blue) of CD39 in presence of estradiol (E2, 1 µM) or vehicle (DMSO) followed by 24-h hypoxia exposure by immunofluorescence. (**B**). Quantification of immunofluorescent images using corrected total cell fluorescence (CTCF) normalized to the number of cells. (**C**). Western blot expression of CD39 in presence of estradiol, fulvestrant (Fulv, 1 µM) or DMSO, vehicle control, following exposure to normoxia (right) or CoCl_2_ (left). (**D**). Western blot of CD39 expression after knockdown of ERα (siESR1) by siRNA. HUVECs were treated with estradiol (E2, 1 µM) or vehicle (DMSO) and exposed to 21% or CoCl_2_ for 24 h. Scrambled control oligos (siCTL) used as negative control. Data represent the mean ± SEM from three independent experiments (black circles). * *p* < 0.05, ** *p* < 0.01; **** *p* < 0.0001 using one-way ANOVA with post hoc Tukey’s test. For 2B: * *p* < 0.05 vs. vehicle control by unpaired *t*-test.

To define the role of hypoxia on the purinergic pathway in HUVECs, we first assessed the expression of CD39 and CD73, two ecto-enzymes responsible for the hydrolysis of ATP to adenosine. Using immunofluorescence, we found a significant increase in CD39 expression with estradiol treatment when compared to DMSO control (Figure 2A,B). No change was seen in CD73 expression levels (Figure S2). To further characterize the estrogen-dependent expression of CD39 in hypoxia, we investigated CD39 expression with pharmacological inhibition of ERα/β (fulvestrant) and ERα-specific inhibition through siRNA (siESR1). Both non-selective and selective inhibition of ERα resulted in a significant decrease in CD39 protein expression (Figure 2C,D). Additionally, CD39 expression was also reduced with the suppression of Specificity Protein-1 (SP1), a transcription factor known to regulate CD39 in hypoxia (Figure S3A) [18]. Previous studies have demonstrated interactions between ERα and SP1 in the transcriptional regulation of target gene promoters [19,20]. It is therefore possible estradiol is contributing to the protective response of endothelial cells through direct or indirect transcriptional regulation of CD39. These results demonstrate the significant role estrogen plays in the regulation of CD39 in hypoxia.

### 2.3. Estradiol-Mediated CD39 Activity Regulates Purinergic Signaling

**Figure 3:**
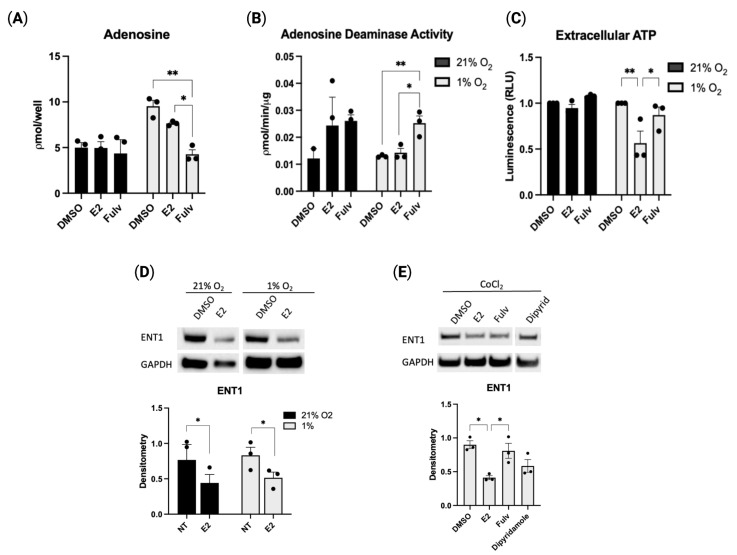
Purinergic pathway activity is affected by estradiol in hypoxic endothelial cells. (**A**). Adenosine concentration, (**B**). Adenosine deaminase (ADA) activity, and (**C**). Extracellular ATP quantified from HUVEC culture media following indicated treatments in 21% or 1% oxygen for 24 h. (**D**,**E**). Representative Western blot showing effect of on ENT1 protein expression following treatment in normoxia or 1% oxygen (**D**) or treatment with cobalt chloride (**E**). Estradiol (E2, 1 µM), fulvestrant (Fulv, 1 µM), dipyridamole (Dipyrid, 5 µM), or vehicle (DMSO). Data represent the mean ± SEM from three independent experiments (black circles). * *p* < 0.05, ** *p* < 0.01 using one-way ANOVA with post hoc Tukey’s test. For 3D: * *p* < 0.05 vs. vehicle control by unpaired *t*-test.

To further define the effect of estrogen on purinergic regulation, we measured various downstream products within the purinergic signaling pathway. Extracellular adenosine, as measured from culture media of HUVECs, is increased in hypoxia. Meanwhile, the inhibition of estrogen receptor activity by fulvestrant decreased the extracellular adenosine concentration (Figure 3A). Adenosine deaminase (ADA), the enzyme responsible for adenosine conversion to inosine, is less active in hypoxia following exposure to E2 when compared to fulvestrant (Figure 3B). Furthermore, in hypoxia, extracellular ATP (eATP) is decreased in the presence of estradiol, while inhibition of ER activity with fulvestrant returns eATP levels to baseline hypoxic levels (Figure 3C). Inhibition of estrogen receptor activity returns adenosine to normoxic levels due to either the previously shown decrease in CD39 and accompanying decrease in ATP hydrolysis, through the increase in ADA activity, or more likely, through both. 

Expression of ENT1, a transmembrane adenosine transport channel that shuttles adenosine bidirectionally, is shown to be significantly reduced with E2 treatment when compared to treatment with DMSO control (Figure 3D); inhibition of estrogen activity restores ENT1 expression to baseline (Figure 3E). Deceased ENT1 increases extracellular adenosine as fewer channels are available for intracellular transport. These combined data establish that treatment with E2 in hypoxic conditions directs purinergic signaling towards increased adenosine production seemingly through increased CD39-mediated ATP hydrolysis and decreased intracellular transport. Increased adenosine is beneficial for endothelial cells as it supports protective hypoxic adaptations, cell integrity, and survival. 

### 2.4. Estradiol Promotes In Vitro Angiogenesis in Hypoxic HUVECs

**Figure 4:**
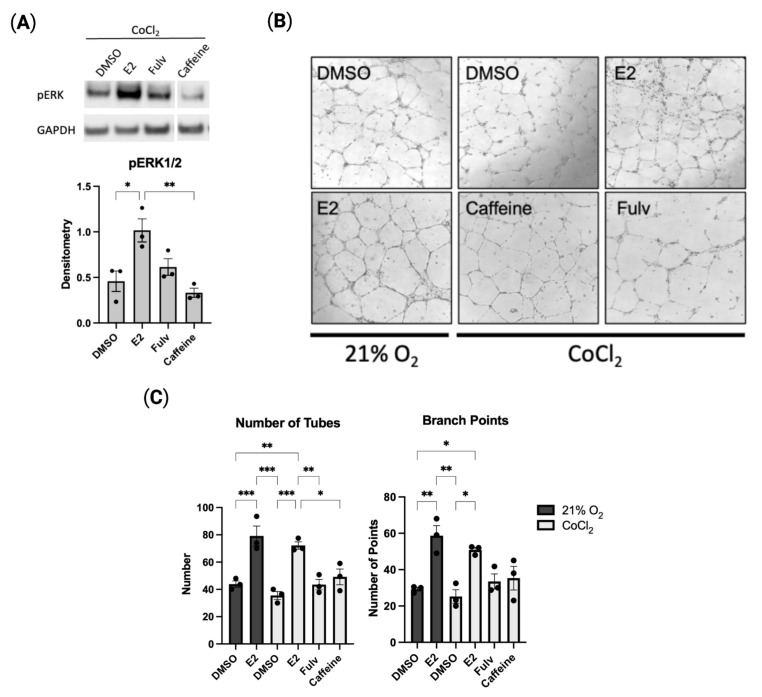
Estradiol promotes in vitro angiogenesis in endothelial cells. (**A**). Representative Western blot showing effect of on pERK1/2 protein expression following treatment in cobalt chloride. (**B**). Representative images of tube formation. HUVECs seeded on basement membrane matrix with indicated treatments and exposed to hypoxia (1% O_2_) for 24 h. (**C**). Number of tube-like structures (left) or number of branching points (right) quantified using ImageJ. Estradiol (E2, 1 µM), fulvestrant (Fulv, 1 µM), caffeine (250 µM) or vehicle (DMSO). Data represent the mean ± SEM from three independent experiments. * *p* < 0.05, ** *p* < 0.01, *** *p* < 0.001 using one-way ANOVA with post hoc Tukey’s test.

Many of the protective effects of adenosine are produced by signaling though adenosine receptors. The alteration in cell signaling associated with these receptors is shown to drive cell proliferation and angiogenesis, among other responses. Extracellular signal-related kinase (ERK) activation has previously been shown to be important in the angiogenic response of adenosine receptor signaling [21]. Levels of phosphorylated ERK (pERK) are increased significantly in HUVECs with E2 exposure compared to control (Figure 4A). ADORA inhibition via caffeine, a known ADORA antagonist, decreased pERK expression. No change was seen in total ERK expression.

The functional effect of estradiol on endothelial cells was further characterized using in vitro models of angiogenesis. In vitro tube formation was evaluated in HUVECs. Estradiol increased the number of capillary-like tubes and branch points in both 21% and 1% oxygen conditions while pharmacological inhibition of ERα/β prevented any increase in tube formation from baseline control (Figure 4B,C). As endothelial cell tube-formation is regulated by many pathways, including adenosine signaling, we next investigated inhibition of the adenosine receptor. Inhibition of ADORA activity also limited the number of tubes and branch points to baseline levels. Similarly, cell migration was measured using the in vitro scratch assay. We have shown that, while the addition of estradiol did not increase wound healing as compared to the control, inhibition of estrogen-mediated responses greatly limited the ability of the endothelial cell to migrate and “heal” the wound (Figure S4). These findings suggest a pro-angiogenic, estrogen-dependent adenosine signaling pathway for hypoxic endothelial cells. 

### 2.5. Estrogen Deficiency Reduces Purinergic Signaling In Vivo

**Figure 5:**
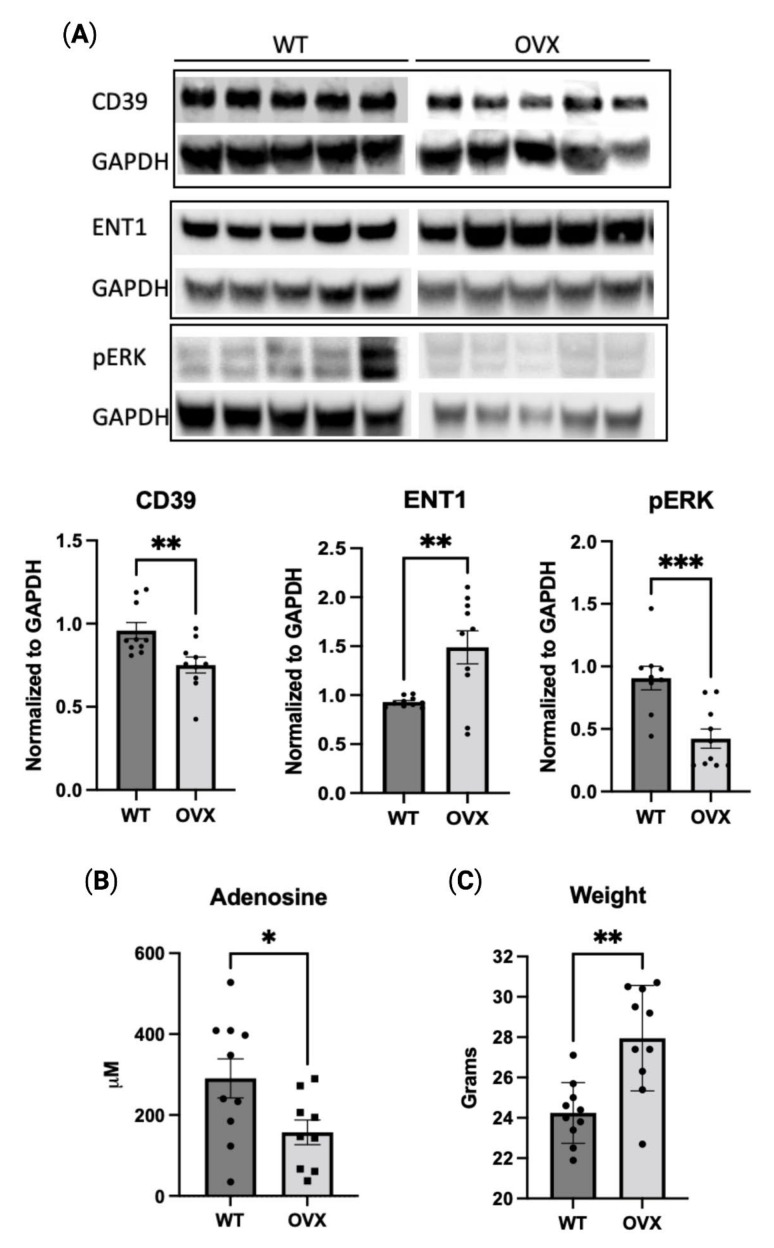
Estradiol mediates purinergic signaling in vivo. (**A**). Representative Western blot of cardiac tissue from WT vs. oophorectomized (OVX) mice for CD39, pERK1/2, or ENT1. (**B**). Adenosine concentration measurement quantified from serum of WT vs. oophorectomized mice. (**C**). Weight of mice at week 24. Data represent the mean ± SEM from each group. *N* = 10/group (black circles). * *p* < 0.05, ** *p* <0.01, *** *p* < 0.001 using unpaired *t*-test.

The estrogen-dependent effects on the purinergic pathway were also assessed in a mouse model of estrogen deficiency. Cardiac tissue from 24-week-old female C57BL/6 mice was analyzed for various markers of purinergic signaling. CD39 expression in cardiac tissue was decreased in the oophorectomized group (OVX) when compared to the wild-type (WT) control (Figure 5A). Additionally, similar to the in vitro results, ENT1 expression was decreased in the OVX group and pERK was increased (Figure 5A). Oophorectomized mice also had decreased levels of serum adenosine (Figure 5B). The serum ADA activity trended upwards in oophorectomized mice (Figure S5). Significant weight gain occurred with oophorectomy alone (Figure 5C). Collectively, these data demonstrate the regulation of purinergic signaling is mediated, at least in part, through the effect of estrogen. 

## 3. Discussion

This study introduces a novel mechanism as to how estradiol protects endothelial cells via the CD39-mediated regulation of the purinergic pathway. Our data show that hypoxic endothelial cells exposed to estradiol significantly increase CD39 expression, leading to positive regulation of adenosinergic signaling and in vitro angiogenesis. Conversely, lack of estrogen activity reversed these effects, both in vitro and in vivo. Previous studies have suggested potential links between estrogen and purinergic signaling, including alterations in the levels of ATP hydrolysis in the platelets of ovariectomized mice and changes in the regulation of ectonucleotidase activity in neurons [22,23]. However, studies delineating the interactions between estrogen and purinergic signaling in the vasculature have been limited. This current study is one of the first to establish a link between estrogen exposure and CD39 regulation in endothelial cells, providing a possible mechanism for vascular protection.

Estrogen is known to regulate microvascular adaptations to injury. Estradiol supports vascular health through stimulation of angiogenesis and vasodilation and through inhibition of perivascular fibrosis and platelet aggregation [9]. Purinergic signaling plays a crucial role in regulating angiogenesis and maintaining microvascular homeostasis following ischemic events. CD39 is the key enzyme involved in converting extracellular ATP to adenosine and has been shown to impact endothelial function, particularly under hypoxic conditions [15]. Overexpression of CD39 has been demonstrated to prevent thrombosis by inhibiting platelet activation, while CD39-null mice have shown higher rates of vascular permeability following ischemia–reperfusion injury [24,25]. CD39 is therefore an important target for developing interventions that can modulate purinergic signaling and improve vascular outcomes.

SP1, a transcription factor commonly associated with HIF-1α, has been shown to regulate transcription of multiple genes in hypoxia including CD39 [18]. SP1 and ERα are also known to cooperate in the transcription of a variety of genes including low-density lipoprotein receptor and adenosine deaminase [20,26]. As such, transcriptional regulation of CD39 in hypoxia is likely mediated through multiple transcription factors including SP1 and ERα. We provide evidence for the hypoxic regulation of ERα and CD39 as well as dependence on estradiol for the increase in expression. Furthermore, reduction in CD39 with suppression of either transcription factor, ERα or SP1, suggest a likely mechanism for such regulation. While further studies are needed to fully elucidate this finding, our data support that the transcriptional regulation of CD39 by ERα is likely through interactions with SP1. 

Adenosine is critical to the microvascular response to injury and hypoxia and has been implicated in endothelial dysfunction. The interaction between adenosine and its receptors initiates intracellular signaling necessary for protective microvascular responses including barrier integrity, remodeling, and inflammatory adaptations [27,28]. Adenosine receptors A2a and A2b are prevalent on endothelial cells and mediate the adenosine response to injury through cAMP/MAPK/ERK pathway signaling [29]. These receptors are specifically regulated by both hypoxia and estrogen, however, crosstalk between adenosine generation and estrogen remains poorly understood [30,31,32]. Our data demonstrate the reduction in adenosine with the inhibition of estrogen receptors and conversely, with estradiol exposure, decreased extracellular ATP. Thus, these findings support that the estrogen-induced changes in CD39 activity led to increased ATP hydrolysis and therefore, increased adenosine production. 

Interestingly, the estrogen-dependent purinergic regulation extends further than just CD39 expression; estradiol is shown here to decrease ENT1 expression and thus, contribute to the prolongation of the protective effects of extracellular adenosine. Hypoxia has previously been shown to regulate expression of ENT1 in HUVECs, presumably as a response to increase extracellular adenosine [33]. However, while Kaneko et al. elegantly demonstrated estradiol inhibition of nucleoside transporter activity in general, the specific regulation of ENT expression by estradiol was previously unknown [34]. Additionally, activation of adenosine receptors frequently results in mitogen-activated protein kinase (MAPK) signal transduction activation. Both hypoxia and estradiol are known to induce ERK activation in endothelial cells; our results demonstrate a synergistic effect between the two, possibly through additive adenosine receptor activity [35,36]. Estrogen-induced adenosine receptor activity is supported by the in vitro angiogenesis assay; no change in tube formation occurs with the inhibition of either estrogen receptor activity or of adenosine receptors. 

The characterization of purinergic signaling in the context of estrogen deficiency in vivo remains relatively limited. Prior studies have indicated that platelet-associated ATP hydrolysis and CD73 activity are substantially impacted following oophorectomy, while adenosine receptor expression elsewhere, as in the brain, is also altered [23,24,37]. Due to the complex interactions between endothelium, myocytes, neurons, and inflammatory cells, we have used whole cardiac tissue to conduct a preliminary exploration of these networks. Our research revealed that oophorectomized mice had lower levels of the purinergic protein, CD39, in cardiac tissue, as well as decreased serum adenosine. Oophorectomy also altered the expression of ENT1 and pERK. Taken together, these findings suggest a role for estrogen in the regulation of purinergic signaling.

The findings presented in this study provide an initial exploration of the intricate interplay between estrogen and purinergic signaling pathways, and therefore, several limitations exist. While this research describes preliminary investigations into the regulation of CD39 in the vasculature by estradiol, further research will be necessary to uncover more connections between these two pathways within the complex signaling system, particularly regarding their in vivo effects. While the study’s findings demonstrate a robust angiogenic effect of estradiol on in vitro angiogenesis, it is likely that the impact of estrogen on angiogenesis is more complex than just the substantive alterations in purinergic signaling. The inhibition of both estrogen and adenosine activity resulted in near complete inhibition of in vitro angiogenesis. We believe that this outcome is, at least in part, the result of estrogen-induced purinergic regulation, considering the other observations of changes in CD39 activity. It is important to note that this study used pooled HUVECs without differentiating between male or female donors, and that future research will include more specific endothelial cell selection, as these cells do not specialize in the microvasculature but express all the proteins of interest.

Given the limitations of estrogen replacement therapy in preventing cardiovascular disease, alternative approaches are needed to achieve effective microvascular modulation [38]. Precisely controlling microvascular function is a complex process, as it involves multiple pathways that can have opposing effects, depending on the underlying pathophysiological states. To develop effective molecular targets for microvascular modulation, it will be crucial to gain better insights into the higher-level regulation of these pathways. Several approaches to CD39 and adenosine receptor modulation are currently under investigation, including small molecule drugs and monoclonal antibodies [39,40]. Many studies targeting the purinergic pathway focus on inhibiting the pathway to limit inflammation. For example, agonist drugs targeting the adenosine receptor have been investigated as potential anti-inflammatory agents for a variety of conditions such as rheumatoid arthritis, asthma, and sepsis [41]. Dipyridamole blocks adenosine transport by ENT and boosts adenosinergic signaling. These effects promote cardioprotective and anti-platelet properties, but these discoveries, as of yet, have not translated into robust clinical benefit [42].

The urgent need remains for the new development of drugs to selectively target those cardiovascular areas at most risk in the setting of a lack of estrogen. The expectation would be to thereby enhance the purinergic pathway to promote angiogenesis, vascular repair, and serve as protective therapies in cardiovascular disease in postmenopausal women.

## 4. Materials and Methods

All research materials are listed in the Major Resources Table File in the Supplementary Materials (Supplementary Tables S1–S5).

*Cell Culture*: Human Umbilical Venous Endothelial Cells (HUVEC) (ATCC, Manassas, VA, USA) were maintained in EBM-2 (Lonza, Cambridge, MA, USA) supplemented with endothelial cell growth media bullet kit (Lonza, Cambridge, MA, USA) on gelatin-coated plastic. For estradiol treatment, cells were maintained in phenol-red free EBM-2 (Lonza, Cambridge, MA, USA) with 2% charcoal-stripped FBS (ThermoFisher, Waltham, MA, USA). Cells were exposed to estradiol (E2) 1 μM (Sigma-Aldrich, St. Louis, MO, USA), fulvestrant (Fulv) 1 μM ((Sigma-Aldrich, St. Louis, MO, USA)), YC-1 10 μM (Enzo Life Sciences, Farmingdale, NY, USA), dipyridamole 5 μM (ThermoFisher, Waltham, MA, USA), and caffeine 250 μM (Sigma-Aldrich, St. Louis, MO, USA) for 24 h, unless otherwise noted. Hypoxia was achieved using 1% oxygen and 5% carbon dioxide or cobalt chloride (CoCl_2_) 100 μM, a hypoxic-mimic known to stabilize HIF-1α, for 24 h (Figure S1A). 

*Immunoblotting:* Cell and tissue lysates were prepared using RIPA buffer (ThermoFisher, Waltham, MA, USA) plus phosphatase inhibitors (Sigma-Aldrich, St. Louis, MO, USA) and cOmplete protease inhibitor cocktail (Sigma-Aldrich, St. Louis, MO, USA) followed by protein quantification with BCA protein assay (Pierce, Rockford, IL, USA). After blocking, membranes were incubated with the following antibodies: CD39 (Invitrogen, Frederick, MD, USA), ERα (Abcam, Waltham, MA, USA), ERβ (Abcam, Waltham, MA, USA), ENT1 (Proteintech, Rosemont, IL, USA). The antibodies against pERK1/2, GAPDH, and HRP-linked anti-rabbit IgG and anti-mouse IgG were purchased from Cell Signaling Technologies (Danvers, MA, USA). Protein was visualized using SuperSignal West Pico PLUS Chemiluminescent Substrate (ThermoFisher, Waltham, MA, USA) on ChemiDoc MP imaging system (Bio-Rad, Hercules, CA, USA) and analyzed with ImageJ software (National Institutes of Health, Bethesda, MD, USA). Band density was normalized using densitometry of glyceraldehyde-3-phosphate dehydrogenase (GAPDH).

*Immunofluorescence*: A total of 24 h after plating, HUVECs were rinsed in PBS, fixed in 2% paraformaldehyde for 10 minutes, and permeabilized with Triton X-100 (ThermoFisher, Waltham, MA, USA). Non-specific proteins were blocked with 7% normal horse serum and incubated with anti-CD39 (1:100; Invitrogen, Frederick, MD, USA) in a humidified chamber at 4 °C overnight. Following washing in PBS, the cells were incubated with Alexa Fluor donkey anti-rabbit secondary antibody (1:600; Invitrogen, Frederick, MD, USA) for 1 hour. Hoechst 33258 (1:10,000; Invitrogen, Frederick, MD, USA) was applied, and after drying, coverslips were placed using Gelvatol. No-primary antibody controls, incubated only with Alexa Fluor secondary antibodies, were included. Images were quantified by corrected total cell fluorescence (CTCF) using ImageJ (National Institutes of Health, Bethesda, MD, USA).

*Gene silencing:* HUVECs were exposed to silencing of *ESR1* and *SP1* using Silencer Select siRNA (ThermoFisher, Waltham, MA, USA). Briefly, cells were resuspended in DMEM without antibiotics (ThermoFisher, Waltham, MA, USA) and seeded at 8 × 10^5^/well in a 6-well plate. Specific siRNAs, including scramble siRNA (siCTL), were incubated with Lipofectamine RNAiMAX (ThermoFisher, Waltham, MA, USA) in Opti-mem to a final concentration of 10 nmol/well. After 24 h, media was changed to phenol-red free EBM-2 media with charcoal-stripped FBS (2%) plus DMSO or E2, and cells were placed in cobalt chloride for an additional 24 h. Knockdown efficiency quantified using qRTPCR (Figure S3B). 

*q-RT-PCR:* CD39, ESR1, and SP1 expression was determined by qPCR. Following total RNA extraction using RNeasy Mini Kit (Qiagen, Beverly, MA, USA) mRNA reverse transcription using MultiScribe Reverse Transcriptase (Invitrogen, Frederick, MD, USA) was completed according to the manufacturer’s instructions. Primer sequences are as follow in Table 1.

Samples were run on CFX Opus 96 Real-Time PCR System (Bio-Rad, Hercules, CA, USA) using SsoAdvanced Universal SYBR Green Supermix (Bio-Rad, Hercules, CA, USA). Results were analyzed by matched software and expressed as relative quantification. Relative gene expression was determined after normalization to GAPDH.

*ATP Assay:* HUVECs were seeded in 96-well plates. Following indicated treatments including 21% or 1% oxygen for 24 h, ATP was measured from cells, media alone, and cells with media using CellTiter-Glo Reagent (Promega, Madison, WI, USA), according to the manufacturer’s protocol. Luminescence was measured at 500 ms in triplicate. Results were normalized to DMSO control.

*Adenosine and Adenosine Deaminase Activity Assay*: HUVECs were plated and incubated with treatments as indicated for 24 h. For adenosine assay, media was collected from cultured HUVECs and immediately snap frozen. Adenosine levels were measured by using Adenosine Assay Kit (Fluorometric; Abcam, Waltham, MA, USA), according to the manufacturer’s protocol. Results were normalized to assay background and quantified using adenosine standard curve. For ADA assay, cells were lysed with ADA assay buffer per the manufacturer’s protocol and immediately snap frozen. Adenosine Deaminase (ADA) activity levels were measured using Adenosine Deaminase Activity Assay Kit (Fluorometric; Sigma-Aldrich, St. Louis, MO, USA) according to the manufacturer’s protocol. Results were normalized to assay background and quantified using inosine standard curve per the manufacturer’s protocol.

*In Vitro Scratch Assay:* HUVECs were seeded in a 24-well culture plate and allowed to form a confluent monolayer. The layer of cells was scraped with a 20–200 μL micropipette tip to create a scratch. Cells were washed with PBS and cultured in phenol-red free EBM-2 with 2% charcoal-stripped FBS media containing indicated treatments. Plates were maintained in 1% oxygen for 24 h. Scratches were imaged at 0- and 24-h using phase-contrast at 4× magnification. Quantification of scratch closure was performed using ImageJ (National Institutes of Health, Bethesda, MD, USA) and expressed as a percentage of the scratched area at time zero. The experiment was performed three independent times.

*Tube Formation Assay:* HUVECs (5 × 10^4^) plated on 24-well plate coated with growth factor reduced basement membrane matrix (Cultrex Basement Membrane Extract; R&D Systems, Minneapolis, MN, USA) in phenol-red free EBM-2 with 2% charcoal-stripped FBS media containing indicated treatments. Tube-like structures were imaged following 24-h hypoxia exposure (CoCl_2_ 100 μM) using phase-contrast at 4× magnification. Quantification of tubes was performed using ImageJ (National Institutes of Health, Bethesda, MD, USA). The experiment was performed three independent times.

*Mice:* Female C57BL/6 mice (n = 10/group) were purchased from and housed at Charles River Labs (Wilmington, MA, USA). Oophorectomy was performed at 12 weeks by Charles River Labs. At week 22, animals were transferred to the animal facilities at BIDMC. At week 24, animals were weighed and euthanized. Whole blood was collected by terminal cardiac puncture, placed in heparin-containing tubes, and centrifuged for plasma separation. Following euthanasia, cardiac tissue was rapidly excised and processed. Tissue lysates were prepared through homogenization of whole cardiac tissue in RIPA buffer. Immunoblot performed as per previously described. The serum used for the measurement of adenosine and ADA activity was per previously described. All studies were approved by the IACUC at BIDMC and adhered to the National Institutes of Health guidelines for the care and use of laboratory animals under the Animal Welfare Act.

*Statistical Analysis:* Results are expressed as mean + SEM. Comparisons were performed using unpaired Student’s *t*-tests. One-way ANOVA, followed by post-test Tukey’s multiple comparison tests, was used when comparing more than 2 sets of data. *p* < 0.05 was considered significant. Statistical analysis was performed using GraphPad Prism, version 9.0 (GraphPad Software, Boston, MA, USA).

## 5. Conclusions

In this study, a novel regulation of purinergic signaling by estrogen in endothelial cells is reported. The upregulation of ERα and induction of CD39 expression by estrogen under hypoxic conditions is demonstrated. This regulation increases extracellular adenosine, which promotes in vitro angiogenesis. The in vivo findings of reduced purinergic responses with estrogen depletion further support these outcomes. Understanding the interactions between CD39, adenosine, and estrogen signaling will improve our knowledge of the effects of hormonal regulation on the cardiovascular system and identify potential targets for future therapeutic intervention.

## Figures and Tables

**Table 1:** Primer sequences.

Primer	Forward	Reverse
CD39	5′ CTGATTCCTGGGAGCACATC 3′	5′ GACATAGGTGGAGTGGGAGAG 3′
ESR1	5′ GAAAGGTGGGATACGAAAAGACC 3′	5′ GCTGTTCTTCTTAGAGCGTTTGA 3′
SP1	5′ TTGAAAAAGGAGTTGGTGGC 3′	5′ TGCTGGTTCTGTAAGTTGGG 3′
GAPDH	5′ GGAGCGAGATCCCTCCAAAAT 3′	5′ GCCTGTTGTCATACTTCTCATGG 3′

## Data Availability

Data are contained within the article and the Supplementary Materials.

## Supplementary Materials

The following supporting information can be downloaded at: https://www.mdpi.com/article/10.3390/ph16030422/s1, Figure S1: Estradiol positively regulates HIF-1α in hypoxia; Figure S2: Estradiol does not change CD73 expression; Figure S3: siRNA mediated reduction of target genes; Figure S4: Endothelial cell migration halted with inhibition of estrogen activity; Figure S5: Serum Adenosine deaminase (ADA) measurement quantified from serum of WT v oophorectomized mice; Major Resource Tables: Table S1: Animals (in vivo studies); Table S2: siRNA; Table S3: Antibodies; Table S4: Cultured Cells; Table S5: Other Major Resources.
