# Supplementary material for: Regulation of Hypoxic–Adenosinergic Signaling by Estrogen: Implications for Microvascular Injury
Source: Pharmaceuticals (Basel). 2023 Mar 10;16(3):422. doi: 10.3390/ph16030422 (PMC10059944; doi:10.3390/ph16030422)
Supplement: Supplementary file 1 [file pharmaceuticals-16-00422-s001.zip › pharmaceuticals-2212217-supplementary.pdf]

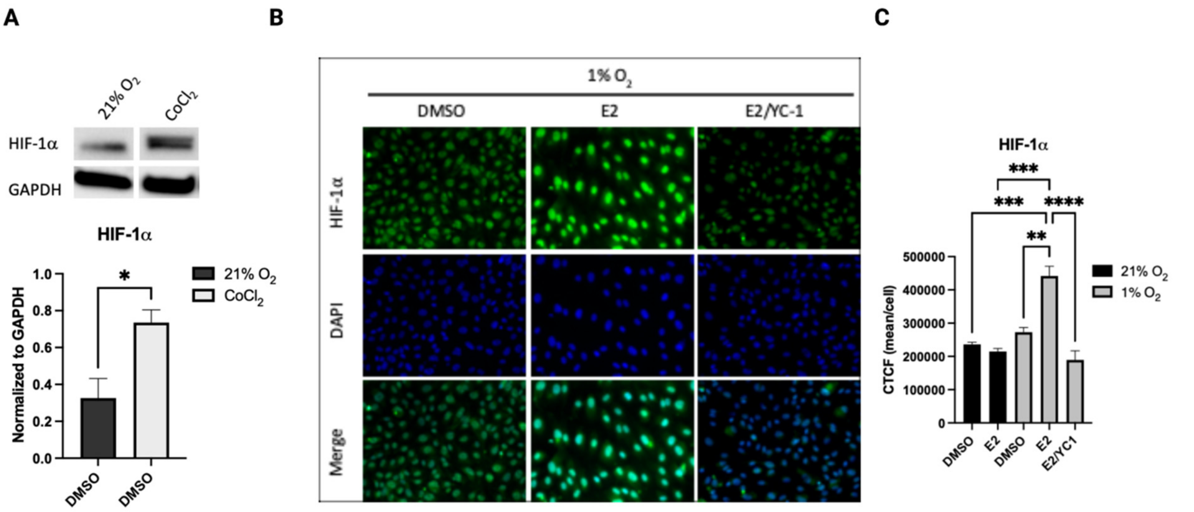

Figure S1. Estradiol positively regulates HIF-1α in hypoxia. (**A**) Representative western blot showing effect of vehicle (DMSO) on HIF-1α protein expression in HUVECs following normoxia (black) or cobalt chloride (gray) treatment. Protein expression quantified with densitometry; (**B**) Representative immunofluorescent images of HUVECs in presence of estradiol (E2, 1μM), YC-1 (10μM) or vehicle (DMSO) followed by 24-hour hypoxia exposure. HIF-1α green, Dapi blue. C. Quantification of HIF-1α immunofluorescence using corrected total cell fluorescence (CTCF) normalized to the number of cells. Data represent the mean ± SEM from three independent experiments. *p <0.05; **p <0.01; ***p <0.001 using one-way ANOVA with *post hoc* Tukey's test. For 1A: *p <0.05 using unpaired t-test.

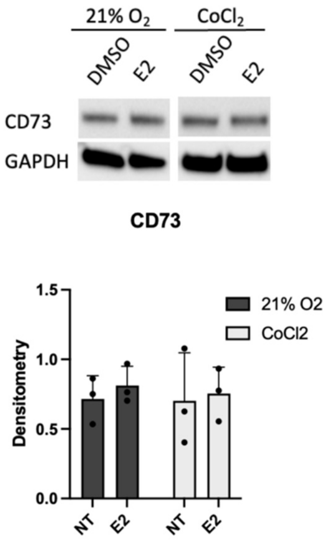

Figure S2. Estradiol does not change CD73 expression. Representative western blot showing effect of vehicle (DMSO) on CD73 protein expression in HUVECs following normoxia (black) or cobalt chloride (gray) treatment. Protein expression quantified with densitometry.

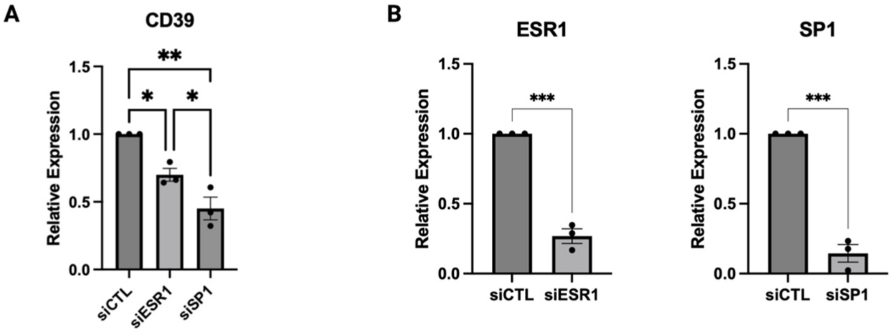

Figure S3. siRNA mediated reduction of target genes. (**A**) Relative gene expression of CD39 detected in HUVECs by q-RT-PCR 48 hours post transfection with siESR1, siSP1, or scramble control, siCTL. HUVECs exposed to $CoCl_2$ for 24 hours; (**B**) Relative gene expression detected by q-RT-PCR 48 hours post transfection with siESR1 (right) or siSP1 (left). Relative expression of targets was calculated by normalizing to expression levels in cells treated with scrambled negative control siRNA (siCTL). Gene expression was determined after normalization to human GAPDH. Data represent the mean ± SEM from three independent experiments. For SF2A: $^{**}p$ <0.01; $^{**}p$ <0.01 using one-way ANOVA with *post hoc* Tukey's test. For SF2B: $^{***}p$ <0.001 using unpaired t-test.

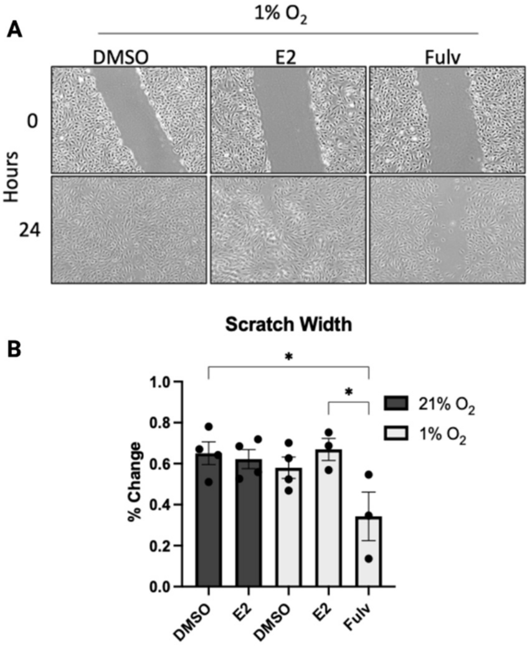

Figure S4. Endothelial cell migration halted with inhibition of estrogen activity. (**A**) Representative images of "wound" at 0 and 24 hours in 1% oxygen; (**B**) Quantification expressed as the percentage change in scratch width over 24 hours. Cells treated with estradiol (E2, 1μM), fulvestrant (Fulv, 1μM), or vehicle (DMSO) and exposed to hypoxia (1% O_2) for 24 hours. Data represent the mean ± SEM from three independent experiments. $^*P$<0.05 using one-way ANOVA with *post hoc* Tukey's test.

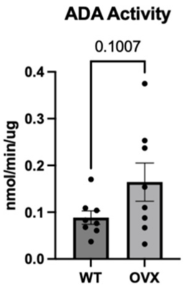

Figure S5. Serum Adenosine deaminase (ADA) measurement quantified from serum of WT v oophorectomized mice. Data represent the mean ± SEM from each group. *N*=10/group. *P*= 0.1007 using unpaired t-test.

Major Resources Table

Table S1: Animals (in vivo studies)

Species	Vendor or Source	Background Strain	Sex	Persistent ID / URL
C57BL/6	Charles River Labs	C57BL/6	F	https://www.criver.com/products-services/find-model/c57bl6-mouse?region=3611#, accessed on 13 January 2022

Table S2: siRNA

Target	siRNA ID	Product Number	Source
SP1	s13319	4392420	Life Technologies
ESR1	s4823	4392420	Life Technologies
Negative Control No. 1 siRNA	AM4611	4392420	Life Technologies

Table S3: Antibodies

Target antigen	Vendor or Source	Catalog #	Working concentration	Lot # (preferred but not required)	Persistent ID / URL
Anti-mouse IgG, HRP-linked	Cell Signaling	7076	1:10,000		RRID:AB_330924
Anti-rabbit IgG, HRP-linked	Cell Signaling	7074	1:20,000		RRID:AB_2099233
CD39	ThermoFisher	MA5-32707	1:1000 (1:100 IF)		RRID:AB_2809984
Donkey anti-Rabbit IgG (H+L) Highly Cross-Adsorbed Secondary Antibody, Alexa Fluor™ Plus 594	Invitrogen	A32754	1:600		RRID:AB_2762827
ENT-1	Proteintech	11337-1-AP	1:1000		RRID:AB_2190784
ERa	Abcam	ab108398	1:1000		RRID:AB_10863604
ERb	Abcam	ab3576	1:1000		RRID:AB_303922
Erk1/2	Cell Signaling	4695	1:1000		RRID:AB_390779
GAPDH	Cell Signaling	2118	1:1000		RRID:AB_561053
Goat anti-Mouse IgG (H+L) Highly Cross-Adsorbed Secondary	Invitrogen	a32723	1:600		RRID:AB_2633275

Antibody, Alexa Fluor™ Plus 488					
HIF-1a	BD Biosciences	610959	1:1000 (1:100 IF)		RRID:AB_398272
phospho-Erk1/2	Cell Signaling	5726	1:1000		RRID:AB_2797617

Table S4: Cultured Cells

Name	Vendor or Source	Sex (F, M, or unknown)	Persistent ID / URL
HUVEC	ATCC	pooled	https://www.atcc.org/products/pcs-100-010, accessed on 4 September 2021

Table S5: Other Major Resources

Description	Source / Repository	Persistent ID / URL
Adenosine Assay Kit (Fluorometric)	Abcam	ab211094
Adenosine Deaminase Activity Assay Kit (Fluorometric)	Sigma	EPI020
BCA Protein Assay	ThermoFisher	23227
Caffeine	Sigma	C0750
CellTiter-Glo® 2.0	Promega	G9242
Charcol Stripped FBS	ThermoFisher	A3382101
Cobalt Chloride	Sigma	15862-1ML-F
cOmplete, Mini, EDTA-free Protease Inhibitor Cocktail	Sigma	11836170001
Cultrex Reduced Growth Factor Basement Membrane Extract, PathClear	R&D Systems	3433-010-01
Dipyridamole	ThermoFisher	J63329.14
DMEM	ATCC	30-2002
DMSO	ThermoFisher	J66650.AE
EGM-2 Endothelial Cell Growth Medium-2 BulletKit	Lonza	CC-3162
Estradiol	Sigma	E2758
Fulvestrant	Sigma	I4409
Hoechst 33258	Invitrogen	H3569
Lipofectamine RNAiMAX Transfection Reagent	ThermoFisher	13778030
MultiScribe Reverse Transcriptase	ThermoFisher	4311235
Optimem	ThermoFisher	31985070
Phosphatase Inhibitor Cocktail Set III	Sigma	524627
RIPA Buffer	ThermoFisher	89901
SsoAdvanced Universal SYBR Green Supermix	Bio-Rad	1725270
SuperSignal West Pico PLUS Chemiluminescent Substrate	ThermoFisher	34579

YC-1	Enzo Life Science	ALX-420-025
